# Cluster Analysis to Identify Possible Subgroups in Tinnitus Patients

**DOI:** 10.3389/fneur.2017.00115

**Published:** 2017-04-03

**Authors:** Minke J. C. van den Berge, Rolien H. Free, Rosemarie Arnold, Emile de Kleine, Rutger Hofman, J. Marc C. van Dijk, Pim van Dijk

**Affiliations:** ^1^Department of Otorhinolaryngology/Head and Neck Surgery, University of Groningen, University Medical Center Groningen, Groningen, Netherlands; ^2^Graduate School of Medical Sciences (Research School of Behavioural and Cognitive Neurosciences), University of Groningen, Groningen, Netherlands; ^3^Department of Neurosurgery, University of Groningen, University Medical Center Groningen, Groningen, Netherlands

**Keywords:** tinnitus, cluster analysis, subgroup identification, heterogeneity of tinnitus, principle component analysis

## Abstract

**Introduction:**

In tinnitus treatment, there is a tendency to shift from a “one size fits all” to a more individual, patient-tailored approach. Insight in the heterogeneity of the tinnitus spectrum might improve the management of tinnitus patients in terms of choice of treatment and identification of patients with severe mental distress. The goal of this study was to identify subgroups in a large group of tinnitus patients.

**Methods:**

Data were collected from patients with severe tinnitus complaints visiting our tertiary referral tinnitus care group at the University Medical Center Groningen. Patient-reported and physician-reported variables were collected during their visit to our clinic. Cluster analyses were used to characterize subgroups. For the selection of the right variables to enter in the cluster analysis, two approaches were used: (1) variable reduction with principle component analysis and (2) variable selection based on expert opinion.

**Results:**

Various variables of 1,783 tinnitus patients were included in the analyses. Cluster analysis (1) included 976 patients and resulted in a four-cluster solution. The effect of external influences was the most discriminative between the groups, or clusters, of patients. The “silhouette measure” of the cluster outcome was low (0.2), indicating a “no substantial” cluster structure. Cluster analysis (2) included 761 patients and resulted in a three-cluster solution, comparable to the first analysis. Again, a “no substantial” cluster structure was found (0.2).

**Conclusion:**

Two cluster analyses on a large database of tinnitus patients revealed that clusters of patients are mostly formed by a different response of external influences on their disease. However, both cluster outcomes based on this dataset showed a poor stability, suggesting that our tinnitus population comprises a continuum rather than a number of clearly defined subgroups.

## Introduction

Tinnitus is a prevalent condition, estimated to affect 5–18% of the adult population (1), which may lead to severe impairment in quality of life. Although many trials on tinnitus therapies have been conducted, hardly ever a treatment effect is demonstrated. A potential explanation for the lack of effectivity of these treatments might be the underlying heterogeneity of the disease. Therefore, consensus on the optimal treatment of tinnitus gradually shifts from a “one size fits all” approach to a more patient-tailored approach. Possibly, a particular group of patients would be more likely to respond to treatment, if a selection is made on etiology, tinnitus characteristics, or patient characteristics. It might be that in a specific subgroup of tinnitus patients a particular treatment is successful, while this treatment is not successful in another subgroup of tinnitus patients. Thus, insight in the heterogeneity of the tinnitus spectrum might improve the management of these patients.

Identification of tinnitus subgroups is also important with regard to concomitant mental distress. Hoekstra et al. demonstrated that patients who express certain characteristics (i.e., high percentage of experience of tinnitus during the day, self-reported depression or anxiety, and subjective experience of tinnitus loudness) are more at risk for a high tinnitus burden (2). This subgroup of patients with high tinnitus distress needs more extensive counseling and follow-up in order to prevent mental breakdown.

In an attempt to identify subgroups of tinnitus patients, cluster analysis was used in this study. Cluster analysis is a statistical technique that divides data into groups, or clusters, which are meaningful and/or useful. It is an explorative analysis that assigns patients to clusters based on certain characteristics, so that patients look very much alike within a cluster (high within-group homogeneity) and, at the same time, are very different from the other clusters (low between-group homogeneity) (3). In research, this cluster analysis method is not only used in medicine studies to identify groups of patients but also in marketing for finding customer segments for example.

In 2008, Tyler et al. performed a preliminary cluster analysis on 153 patients with tinnitus (4). The cluster analysis of Tyler et al. identified distinct cluster characteristics, which were described as: (1) “constant distressing tinnitus,” (2) “varying tinnitus that is worse in noise,” (3) “tinnitus patients who are copers and whose tinnitus is not influenced by somatic modulation,” and (4) “tinnitus patients who are copers but whose tinnitus is worse in quiet environments.” Tyler et al. did not report a statistic value to identify the degree to which patients are clustered in these groups.

In this paper, we report on an exploratory cluster analysis of patients from the tinnitus database of the University Medical Center Groningen (*n* = 1,783 patients). We initially attempted to replicate the cluster analysis reported by Tyler et al (4); however, this was not possible as many of the variables used in their analysis were not identical or not available in our database. Instead, we report on two further cluster analyses. In the first analysis, the choice of variables that were entered in the cluster analyses was fully guided by the statistical techniques. In the second analysis, the selection of variables was based on the expert opinions in our tinnitus clinic. The aim of this study was to identify subgroups of tinnitus using cluster analysis, based on a very large dataset of tinnitus patients.

## Materials and Methods

### Tinnitus Population

This study was performed at the Otorhinolaryngology Department of the University Medical Center Groningen (The Netherlands), which has a specialized multidisciplinary care group for tinnitus patients since 2007. Patients with severe complaints of tinnitus can be referred to this care group for medical consultation and psychological support. Almost all patients who visit this care group have consulted an audiologist and/or otorhinolaryngologist earlier. However, these patients were referred to our specialized tertiary care group by these specialists, because of the severity and impact of the complaints. Consultation at our clinic consists of thorough evaluation by an otorhinolaryngologist, an audiologist, radiologist, a medical social worker, and/or a psychologist.

### Variables

The variables that were available for this cluster analysis were demographic characteristics (e.g., sex and age), tinnitus characteristics (e.g., duration of tinnitus, onset, lateralization, pitch, variable loudness), factors of influence on patients tinnitus (e.g., influence of loud sounds, noisy environment, movement of head and neck), tinnitus and quality of life-related questionnaires [e.g., tinnitus handicap index (THI), visual analog scale (VAS), and hospital anxiety and depression scale (HADS)], and audiological characteristics [e.g., frequency matching, pure tone audiometry (PTAs), loudness matching of tinnitus]. Hearing loss was divided into categories based on the pure tone audiogram: (1) no or slight hearing loss (both ears thresholds <30 dB on PTA thresholds at 0.25–0.5–1–2–4–8 kHz), (2) asymmetrical hearing loss (≥30 dB difference between both ears on the mean PTA thresholds at 2–4–8 kHz), (3) bilateral high tone hearing loss (both ears thresholds ≥30 dB on PTA thresholds at 2–4–8 kHz), (4) bilateral severe hearing loss (PTA thresholds >30 dB on 0.25–0.5–1–2–4–8 kHz), and (5) others. The available variables are all listed in Table S1 in Supplementary Material. All patient-reported variables were completed by the patients in booklets during the visit at the tinnitus outpatient clinic. Physician reported data, such as audiological characteristics, were also reported in booklets by the physician. All these routinely collected data were anonymized and entered in a database. For the current analysis, these data were retrospectively analyzed. The collection of data was approved by the Institutional Reviewer Board of the UMCG. No full review was needed due to the retrospective nature of this study.

### Selection of Variables for Cluster Analysis

All variables that were collected were entered in the database. However, not all of these variables could be entered in the cluster analysis. In cluster analysis, it is important to keep the sample size in mind when deciding how many variables to enter in the analysis. Formann recommends the number of variables (*m*) of 2*^m^* = sample size (5). In our study, the sample size is *n* = 1783, implying that the number of variables should be 10 or 11. There are two ways to select appropriate variables for cluster analysis: (1) a statistical approach with the use of principal component analysis (PCA) and (2) selection of variables based on “expert opinion,” i.e., variables that are presumed to be clinically relevant and thought to be discriminative in the total group. Both selection procedures were performed in this study, resulting in two different cluster analyses.

#### Variable Reduction by PCA

A PCA is a dimension reduction technique that condenses variables that are highly correlated into a set of factors, thereby removing overlap and redundancy. PCA with Varimax rotation was performed on all variables with missing values ≤20%. The PCA revealed several factors, and for each factor, the variable with the highest loading was selected for inclusion in the cluster analysis.

#### Variable Reduction by “Expert Panel”

After excluding variables with missing values >20%, variables were selected by a group of tinnitus care professionals and investigators [Minke J. C. van den Berge (otolaryngology resident, Ph.D. candidate in tinnitus research), Pim van Dijk (medical physicist, audiologist, involved in the tinnitus care group), and Emile de Kleine (medical physicist, audiologist, involved in the tinnitus care group)]. Based on clinical experience and knowledge, those variables were selected that were deemed important in discriminating subgroups of tinnitus.

### Cluster Analysis

The “two-step” cluster analysis method was used as the analyses that contained both categorical and continuous variables (6). Continuous variables were standardized by default. For distance measures, the log-likelihood method was used, as both continuous and categorical variables were entered in the analysis. The number of clusters to be formed was not specified in advance. The “silhouette measure of cohesion and separation” is a measure for the overall goodness-of-fit of the cluster structure that was found. It ranges from −1 to 1 (<0.25: no substantial structure; 0.26–0.50: weak structure and could be artificial; 0.51–0.70: reasonable structure; 0.71–1.0: strong structure) (7).

Differences in characteristics between clusters were compared according to the cluster membership variable, using one-way ANOVA for continuous variables and Pearson Chi-square tests for categorical variables. SPSS version 23.0 (Chicago, IL, USA) was used for all tests. The significance level was set at α = 0.05, and all tests were two tailed.

## Results

### Subject Characteristics

For this study, data from 1,783 consecutive patients who visited the UMCG tinnitus clinic between July 2007 and June 2016 were collected. The baseline characteristics of this study population are shown in Table 1. Variables that had >20% missing values are not shown in this table. In this population, 39.3% were females and the mean age was 53.6 ± 13.5 years. Tinnitus was unilateral in 50.7% of the cases and bilateral or central in 48.2%. The mean THI in the total patient group was 42.5 ± 23.2.

**Table 1:** **Demographic and tinnitus-related characteristic of included tinnitus patients (*n* = 1,783)**.

	Total *N*	
**Demographic characteristics**		
Mean age ± SD	1,783	53.6 ± 13.5
Female gender—no. (%)	1,783	701 (39.3)
**Tinnitus characteristics**		
Mean duration of tinnitus ± SD—in years	1,635	6.8 ± 8.7
Onset tinnitus—no. (%)	1,685	
Acute		813 (48.2)
Gradual		872 (51.8)
Lateralization of tinnitus—no. (%)	1,496	
Bilateral/central		738 (49.3)
Unilateral		758 (50.7)
Description of tinnitus—no. (%)	1,607	
Tonal		715 (44.5)
Noise		708 (44.1)
Other		184 (11.4)
Experience of tinnitus—no. (%)	1,645	
Continuous		1,512 (91.9)
Intervals		133 (8.1)
Pitch of tinnitus—no. (%)	1,620	
Low		79 (4.9)
Moderate		403 (24.9)
High		997 (61.5)
Other		141 (8.7)
Variable loudness of tinnitus—no. (%)	1,762	
Yes		1,271 (72.1)
No		491 (27.9)
Mean percentage of burden during awake time ± SD	1,671	74.7 ± 28.0
Preference for silence or noise—no. (%)	1,559	
Silence		697 (44.7)
Noisy environment		862 (55.3)
Highest burden at time of the day—no. (%)	1,522	
Waking up		131 (8.6)
Morning		38 (2.5)
Afternoon		34 (2.2)
Evening		282 (18.5)
Night		149 (9.8)
Other		888 (58.3)
Sound unpleasant—no. (%)	1,446	
Never		137 (9.5)
Seldom		192 (13.3)
Some times		590 (40.8)
Most of the time		357 (24.7)
Always		170 (11.8)
**Factors of influence on tinnitus**		
Influence of noisy background—no. (%)	1,551	
Tinnitus louder		212 (13.7)
No effect		753 (48.5)
Tinnitus less loud		586 (37.8)
Influence of loud sounds—no. (%)	1,539	
Tinnitus louder		693 (45.0)
No effect		523 (34.0)
Tinnitus less loud		323 (21.0)
Influence of movement of head and neck—no. (%)	1,559	
Tinnitus louder		479 (30.7)
No effect		995 (63.8)
Tinnitus less loud		85 (5.5)
Influence of nap in the afternoon—no. (%)	1,419	
Tinnitus louder		233 (16.4)
No effect		1,003 (70.7)
Tinnitus less loud		183 (12.9)
Influence of stress—no. (%)	1,508	
Tinnitus louder		937 (62.1)
No effect		552 (36.6)
Tinnitus less loud		19 (1.3)
Influence of sleep deprivation—no. (%)	1,491	
Tinnitus louder		847 (56.8)
No effect		629 (42.2)
Tinnitus less loud		15 (1.0)
**Audiological characteristics**		
Mean PTA over 1–2–4 kHz (mean ± SD)	1,764	29.7 ± 18.0
Difference in mean PTA over 1–2–4 kHz between both ears (mean ± SD)	1,764	11.5 ± 18.1
Frequency matching of tinnitus—no. (%)	1,469	
0–2,000 Hz		378 (24.0)
2,000–4,000 Hz		239 (15.5)
4,000–6,000 Hz		287 (18.6)
6,000–8,000 Hz		275 (17.9)
>8,000 Hz		290 (23.4)
Type of hearing loss—no. (%)	1,782	
No/slight hearing loss		989 (55.5)
Asymmetrical hearing loss		265 (14.9)
Bilateral high tone hearing loss		243 (13.6)
Bilateral severe hearing loss		246 (13.8)
Other		39 (2.2)
**Tinnitus questionnaires**		
VAS tinnitus loudness^a^ (mean ± SD)	1,615	66.7 ± 20.9
VAS tinnitus annoyance^a^ (mean ± SD)	1,641	69.1 ± 22.6
THI-score (mean ± SD)	1,505	42.5 ± 23.2
HADS-depression score ± SD	1,676	5.4 ± 4.3
Indication HADS-depression^b^—no. (%)	1,676	
No indication depression		1,321 (78.8)
Indication depression		355 (19.9)
HADS-anxiety score ± SD	1,690	6.9 ± 4.3
Indication HADS-anxiety^b^—no. (%)	1,690	
No indication anxiety		1,103 (65.3)
Indication anxiety		587 (34.7)

*^a^On visual analog scale (VAS) range from 0 to 100%*.

*^b^Range 0–21, indication for depression/anxiety with score >8*.

*Range is 0–100 unless indicated otherwise*.

*dB, decibel; PTA, pure tone audiometry; THI, tinnitus handicap inventory; HADS, hospital anxiety and depression scale*.

### Outcome of Cluster Analysis with Variables Selected by PCA

The PCA was performed to obtain eigen values for each factor. The Kaiser–Meyer–Olkin measure of sampling adequacy was 0.681 and the Bartlett’s test of sphericity was significant [χ^2^ (6,127) = 325, *p* < 0.001], both indicating an appropriate factor model. A total of eight factors were extracted (based on the eigenvalue >1 rule), which together explained 55% of the total variance. Variables with the highest loading on each factor were selected. Subsequently, these variables (*n* = 8) were entered in the cluster analysis. The clustering revealed a four-cluster solution. As the analysis excludes every case when there is any variable with a missing value (listwise exclusion), the analysis was based on *n* = 976 patients. The cluster outcome showed a “silhouette measure of cohesion and separation” of 0.20, indicating that it is a “no substantial” cluster solution (7). Characteristics of these four identified clusters are shown in Table 2. The variables in the table are ranked from most discriminative between groups (top of the table) to less discriminative (bottom of the table). All variables differed statistically significant between the four clusters, except for the variables “VAS tinnitus annoyance” and “frequency of the tinnitus” (*p* = 0.925 and *p* = 0.478, respectively).

**Table 2:** **Characteristics of the four clusters identified by clustering with variable selection based on principal component analysis**.

	Cluster 1 (*n* = 293)	Cluster 2 (*n* = 259)	Cluster 3 (*n* = 197)	Cluster 4 (*n* = 227)	*p*-Value
Influence of loud sound (%)					**<0.001^a^**
Tinnitus louder	37.5	54.4	0	68.7	
No effect	62.5	39.4	0	30.0	
Tinnitus less loud	0	6.2	100	1.3	
Influence of sleep deprivation (%)					**<0.001^a^**
Tinnitus louder	0	89.2	47.2	86.8	
No effect	100	10.8	49.7	11.5	
Tinnitus less loud	0	0.8	3.0	1.8	
Onset (%)					**<0.001^a^**
Acute	48.1	0	49.2	100	
Gradual	51.9	100	50.8	0	
Influence of nap afternoon (%)					**<0.001^a^**
Tinnitus louder	0	27.0	11.2	22.9	
No effect	100	56.8	84.3	52.4	
Tinnitus less loud	0	16.2	4.6	24.7	
Hospital anxiety and depression scale (mean)	4.6	5.6	5.5	6.0	**0.001^b^**
Difference in mean PTA ADS	13.1	10.8	7.9	11.9	**0.015^b^**
Frequency of tinnitus (%)					0.478^a^
0–2,000 Hz	23.2	23.2	21.8	25.1	
2,000–4,000 Hz	14.7	13.9	21.3	15.0	
4,000–6,000 Hz	18.8	20.5	20.3	15.0	
6,000–8,000 Hz	19.5	17.0	12.7	19.4	
>8,000 Hz	23.9	25.5	23.9	25.6	
Visual analog scale tinnitus annoyance (mean)	69.2	69.4	68.2	69.6	0.925^b^

*^a^Pearson Chi-square test*.

*^b^One-way ANOVA*.

*ADS, both ears; PTA, pure tone audiometry*.

*Bold fonts represent a significant p-values (p < 0.05)*.

Cluster 1 (*n* = 293) is characterized by the fact that tinnitus is not easily influenced: loud sounds, sleep deprivation, and nap in the afternoon have no effect on their tinnitus. These patients have a relatively high difference between hearing loss in the right and left ear. These patients have relatively low HADS-depression scores.

Cluster 2 (*n* = 259) is distinguished by a gradual onset of the tinnitus. Also in this group, tinnitus is easily negatively influenced, especially by loud sounds and sleep deprivation. Both make their tinnitus louder.

Cluster 3 (*n* = 197) is a group of patients who report that their tinnitus is less loud when they hear loud sounds. Sleep deprivation and a nap in the afternoon mostly have no effect on their tinnitus.

Cluster 4 (*n* = 227) is typically a group with tinnitus of acute onset. They report that their tinnitus is easily negatively influenced by loud sounds or sleep deprivation. They show relatively high HADS-depression scores.

### Outcome of Cluster Analysis with Variables Selected by Expert Panel

For the alternative method of choosing variables for clustering, 11 variables were selected by a panel of experts in the field. The selected variables (see Table 3) were entered in the cluster analysis. The outcome was a three-cluster solution, with a “silhouette measure of cohesion and separation” of 0.20, again indicating a poor solution. Because of listwise exclusion as described earlier, this analysis was based on *n* = 761 patients. About 527 of these patients were also included in the first cluster analysis. Also in this table, variables are ranked according to their degree of discriminative value. All variables differed significantly between the clusters (all *p*-Values <0.001).

**Table 3:** **Characteristics of the four clusters identified by clustering with variables selected by expert opinion**.

	Cluster 1 (*n* = 287)	Cluster 2 (*n* = 247)	Cluster 3 (*n* = 227)	*p*-Value
Influence of loud sound (%)				**<0.001^a^**
Tinnitus louder	16.0	27.9	96.9	
No effect	56.1	38.5	3.1	
Tinnitus less loud	27.9	33.6	0	
Influence of stress (%)				**<0.001^a^**
Tinnitus louder	24.4	87.4	78.4	
No effect	74.9	9.7	21.6	
Tinnitus less loud	0.7	2.8	0	
Preference for silence or noise (%)				**<0.001^a^**
Silence	28.2	25.5	89.9	
Noisy environment	71.8	74.5	10.1	
Are sounds uncomfortably loud? (%)				**<0.001^a^**
Never	18.8	6.9	0.4	
Seldom	28.9	13.4	3.1	
Sometimes	31.4	61.5	31.7	
Most of the time	14.6	13.4	43.2	
Always	6.3	4.9	21.6	
Lateralization of tinnitus (%)				**<0.001^a^**
Bilateral/central	31.0	84.2	38.3	
Unilateral	69.0	15.8	61.7	
Hearing loss category (%)				**<0.001^a^**
No hearing loss	51.9	72.5	44.9	
Asymmetrical hearing loss	15.7	2.4	35.2	
Bilateral high tone hearing loss	17.4	8.9	9.7	
Bilateral flat hearing loss	12.9	16.2	6.2	
Other	2.1	0	4.0	
Variable loudness (%)				**<0.001^a^**
No	49.8	15.0	18.1	
Yes	50.2	85.0	81.9	
Tinnitus handicap index-score (mean)	32.6	48.0	46.9	**<0.001^b^**
Influence of movement of head and neck (%)				**<0.001^a^**
Tinnitus louder	8.4	40.1	39.2	
No effect	84.3	55.5	55.9	
Tinnitus less loud	7.3	4.5	4.8	
Hospital anxiety and depression scale (mean)	4.2	5.9	5.4	**<0.001^b^**
Gender (%)				**<0.001^a^**
Male	59.9	74.1	55.9	
Female	40.1	25.9	44.1	

*^a^Pearson Chi-square test*.

*^b^One-way ANOVA*.

*Bold fonts represent a significant p-values (p < 0.05)*.

Cluster 1 (*n* = 287) is a group of patients whose tinnitus is not easily influenced: loud sounds, stress, or movement of head and neck have no effect on their tinnitus loudness. Patients prefer a noisy environment. Sounds are never to seldom experienced as uncomfortably loud. The tinnitus is mostly unilateral. Although most patients in this group have no or slight hearing loss, other types of hearing loss are present in this group as well. They are not very much bothered or depressed by their tinnitus, as the THI and HADS-depression scores are low.

Cluster 2 (*n* = 247) is a predominantly male group, whose tinnitus gets worse by stress, loud sounds, and movement of head and neck. These patients prefer to be in a noisy environment. Sometimes, sounds are experienced as uncomfortably loud. Most of the patients have no or slight hearing loss. Tinnitus is bilateral, and the loudness of the tinnitus is variable.

Cluster 3 (*n* = 227) is characterized by the fact that their tinnitus is easily negatively influenced: loud sounds and stress clearly make their tinnitus louder. These patients prefer a silent environment. Often, patients find sounds uncomfortably loud. Tinnitus is often bilateral with most patients having no or slight hearing loss or asymmetrical hearing loss. The loudness of the tinnitus is variable.

## Discussion

### Key Results

In this study, we performed cluster analysis with the aim to identify subgroups in a population of tinnitus patients. Variable selection for cluster analysis was performed in two ways: by a strict methodological approach based on PCA, and by expert opinion. These analyses identified four- and three-patient clusters, where the clusters showed clearly different characteristics. However, the clustering solution in both analyses was not substantial, as indicated by a poor cluster solution quality.

Although both cluster analyses gave different outcomes, there were also interesting similarities. In both cluster solutions, the effects of “stress” and “loud sounds” on tinnitus have a relatively high discriminative value between groups. In each analysis, a group was revealed in which patients report that their tinnitus gets louder from loud sounds, and there was a group that reported that their tinnitus got less loud. In an earlier cluster analysis by Tyler et al., it is described that their found clusters differed by the effect of external factors on patients’ tinnitus: some patients are easily and negatively influenced by external factors and, in others, this has no effect (4). On the contrary, Tyler et al. describe a group that is characterized by high scores on tinnitus questionnaires and the HADS depression and anxiety scale. However, this was not reflected in our cluster solutions.

In the cluster analysis based on variables selected by experts, there was a clear distinction between a group that preferred a silent environment for their tinnitus and another group that had a preference for a noisy environment. The fact that some patients with tinnitus prefer noise and others prefer silence has been described earlier (8). This is interesting, as one might speculate that the latter group may have a higher change of responding well to sound therapy than the other group.

When interpreting these results, it must be kept in mind that the “silhouette measure” of both analyses was only 0.2. This is lower than the critical boundary of 0.25, which implies that there was no substantial clustering in this patient cohort. A lack of clustering indicates that the transition from one cluster to another is relatively smooth, without clear-cut boundaries. As a comparison, consider a group of cities, where the coordinates of the cities would go into a cluster analysis. If one group of cities is clearly separated from another group by a stretch of open land, the silhouette value will be large (when viewed from a distance, the cities will have a distinct silhouette of their skyline). However, if there is no such open land between the clusters, the silhouette value is low, consistent with the absence of substantial clustering. In our patient cohort, there were clearly no distinct “open stretches of land” between the clusters, suggesting that patient form a continuum rather than a clear clustering. As discussed above, the cluster analysis of Tyler et al. identified clusters with characteristics that show some resemblance to the clusters reported here. Unfortunately, Tyler et al. do not report a silhouette value or other measure of clustering. Hence, at present, it is not possible to discuss the clustering strength in their cohort.

Cluster analysis has been upcoming in medical research. Recently, an interesting cluster analysis on bilateral Meniere disease was published to define clinical subgroups with potential similar etiologies. In this study, five clinical variants of bilateral Meniere disease were found based on six clinical variables and with a high silhouette measure of 0.8 (9). This study is not only beneficial to improve the selection of patients but also can explain the negative treatment effects of several treatment trials, as results can be biased by a heterogeneous patient group based on etiology (9). The difficulty in cluster analysis is that it is a type of analysis that is very sensitive to change of variables. The selection of variables is critical for the outcome of the cluster analysis (6). Generally, highly correlated variables should be avoided and it is important to select variables that can make a clear-cut differentiation between clusters (6). The systematic statistical approach of selecting variables using the highest factor loading on extracted factors by PCA is often used and has the advantage of choosing variables in a reproducible, transparent way. A downside of this technique is that the factor solution only explains a certain amount of variance and, therefore, much information is discarded. Eliminating factors with low loadings on the extracted factors has the same effect (10). This may lead to a reduced success of a subsequent cluster analysis. On the other hand, a disadvantage of selecting variables based on clinical knowledge or “gut feeling” is that it is less transparent. Also, unrecognized highly discriminating variables may remain undiscovered.

### Strengths of the Study

For this study, a very large database of tinnitus patients was used with almost 1,800 patients. Even after exclusion of patients with missing values, *n* = 976 and *n* = 761 could be included in the cluster analyses. We expect that, if clear clustering would have existed with these variables, we would have been able to find it in these groups. There was an overlap of 527 patients who were included in both the first cluster outcome and the second cluster outcome. This is a substantial overlap, pointing out that it does not seem likely that the differences between both cluster analyses are caused by the differences in included patients.

### Limitations

This study explored the patient cohort of a tertiary tinnitus referral center. Thus, the population described here consists of a group of tinnitus patients who were persistent in their search for treatments for their tinnitus. Our patient cohort may, therefore, be biased with a certain type of tinnitus patients. Potentially, a study including also less-persistent help seeking and non-help-seeking subjects would have identified a clearer clustering.

Although we had access to a large database of tinnitus patients (*n* = 1,783), over the years, there were changes in the variables that were collected because of changes made to the diagnostic protocol. Since the cluster analyses required a complete set of data for each patient, not all 1,783 patients could be included in the analysis, but 976 and 761.

Furthermore, it is debated internationally whether tinnitus is a disease or a symptom. One can look at it in both ways: when tinnitus is a result of an acoustic neuroma, then tinnitus can be a symptom. However, if we look at tinnitus as the result of defect on a cellular level of the auditory cortex, then tinnitus can be regarded as a disease. In most patients visiting our clinic, the etiology of the tinnitus is unclear. The fact is that these patients included in our dataset experience bothersome tinnitus. Within this group, we aimed to find subgroups such as patients with continuous central, loud tinnitus tend to have a high score on THI and VAS and find that their tinnitus gets worse in noisy environments. If we are able to find such patterns, may be we can adjust our treatment strategy to that (in this example, hearing aids might not be successful). Although the raised issue about tinnitus being a symptom or disease is important, we believe that this analysis looking for clusters of patients based on tinnitus characteristics transcends this issue.

Finally, the low silhouette value indicates that this patient cohort represents a heterogeneous group without clear clustering. Obviously, any cluster analysis outcome highly depends on the variables that we entered into the clustering algorithm. Our patient data consisted of mainly audiometry and questionnaire metrics. In these cluster analyses, tinnitus patients appear to represent a continuum rather than clearly defined subgroups, based on a low silhouette measure. However, it is possibly that other metrics (e.g., fMRI/EEG, genetic evaluation) are able to identify tinnitus subgroups. In other words, the lack of clustering in our analyses does not imply that clusters do not exist. However, if clusters exist, they cannot be identified with the variables that were considered here.

## Conclusion

Two cluster analyses of a large patient cohort identified three and four groups of tinnitus patients, respectively. The clustering was not substantial, as a low silhouette measure of the cluster solutions was found. This indicates that in this particular cohort, tinnitus patients appear to represent rather a continuum than clearly defined subgroups. This finding may have consequences for future treatments: if clear subgroups would have been present, clearly distinct treatment might be developed in the future. However, for a continuum of patients, it may be necessary to use a number of treatments to find the optimum for each individual patient. Obviously, our conclusion is based on the set of variables that were at our disposal. Possibly, new future ways to characterize tinnitus patients may be able to find distinct subgroup in tinnitus patients.

## Author Contributions

Conception of study and design; writing of manuscript: MB and PD. Intellectual contribution to analyses: MB, PD, and EK. Critical revision of the manuscript: RF, RA, PD, and JD. Data acquisition: MB, PD, RA, RH, and EK.

## Conflict of Interest Statement

The authors declare that the research was conducted in the absence of any commercial or financial relationships that could be construed as a potential conflict of interest. The reviewer CM and handling Editor declared their shared affiliation, and the handling Editor states that the process nevertheless met the standards of a fair and objective review.
