# Supplementary material for: Cluster Analysis to Identify Possible Subgroups in Tinnitus Patients
Source: Front Neurol. 2017 Apr 3;8:115. doi: 10.3389/fneur.2017.00115 (PMC5377919; doi:10.3389/fneur.2017.00115)
Supplement: Supplementary file 1 [file Data_Sheet_1.DOCX]

Supplementary Material

Cluster analysis to identify subgroups in tinnitus patients

M.J.C. van den Berge^1^, R.H. Free^1^, R. Arnold^1^, E. de Kleine^1^, R. Hofman^1^, J.M.C. van Dijk^2^, P. van Dijk^1^

*** Correspondence:** M.J.C. van den Berge: m.j.c.van.den.berge@umcg.nl

**Table 1.** List of available variables

|  | **Variable name** | **Source of data** | **Data type** | **Description of variable** |
| --- | --- | --- | --- | --- |
| **Demographics** | **Gender** | **PR** | **Binary** | **Male/Female** |
|  | **Age** | **DD** | **Continuous** | **In years** |
| **Tinnitus characteristics** | **Duration of tinnitus** | **PR, DD** | **Continuous** | **In years** |
|  | **Onset of tinnitus** | **PR** | **Binary** | **Acute / Gradual** |
|  | **Lateralization of tinnitus** | **PR** | **Categorical** | **Unilateral / Bilateral or Central** |
|  | **Description of tinnitus** | **PR** | **Categorical** | **Tonal / Noise / Other** |
|  | **Experience of tinnitus** | **PR** | **Binary** | **Continuous / Intervals** |
|  | **Pulsating tinnitus** | **PR** | **Categorical** | **Not pulsating / Pulsating synchronous with heartbeat / Pulsating not synchronous with heartbeat** |
|  | **Pitch of tinnitus** | **PR** | **Categorical** | **Low / Moderate / High / Other** |
|  | **Variable loudness of tinnitus** | **PR** | **Binary** | **Yes / No** |
|  | **Percentage of burden during awake time** | **PR** | **Continuous** | **Range 0-100%** |
|  | **Preference for silence or noise** | **PR** | **Binary** | **Silence / Noisy environment** |
|  | **Daytime with highest burden** | **PR** | **Categorical** | **Awaking / Morning / Afternoon / Evening / Night / Other** |
|  | **Is sound unpleasant?** | **PR** | **Categorical** | **Never / Seldom / Sometimes / Most of the time / Always** |
| **Audiological characteristics** | **Etiology of hearing loss** | **CR** | **Categorical** | **Presbyacusis / Noise exposure / Otosclerosis / Congenital / Otitis Externa /**  **ISSHL / OME/OMA / Cholesteatoma /**  **Lyme disease / Other or Unknown** |
|  | **Frequency matching** | **CR** | **Categorical** | **0-2000 Hz / 2000-4000 Hz / 4000-6000 Hz / 6000-8000 Hz / >8000 Hz** |
|  | **Loudness matching** | **CR** | **Continuous** | **In dB** |
|  | **Fletcher Index Right / Left ear** | **CR** | **Continuous** | **In dB (mean thresholds 2-4-8 kHz)** |
|  | **Difference in Fletcher Index between right and left ear** | **CR, DD** | **Continuous** | **In dB** |
| **Tinnitus Questionnaires** | **VAS tinnitus loudness** | **PR** | **Continuous** | **Range 0-100** |
|  | **VAS tinnitus annoyance** | **PR** | **Continuous** | **Range 0-100** |
|  | **THI-score** | **PR, DD** | **Continuous** | **Range 0-100** |
|  | **HADS-depression** | **PR, DD** | **Continuous** | **Range 0-21** |
|  | **HADS-anxiety** | **PR, DD** | **Continuous** | **Range 0-21** |
|  | **HQ-score** | **PR, DD** | **Continuous** | **Range 0-42** |
| **Influences on tinnitus** | **Influence of noisy background** | **PR** | **Categorical** | **No effect / Tinnitus less loud / Tinnitus louder** |
|  | **Influence of movement of head and/or neck** | **PR** | **Categorical** | **No effect / Tinnitus less loud / Tinnitus louder** |
|  | **Influence of nap in the afternoon** | **PR** | **Categorical** | **No effect / Tinnitus less loud / Tinnitus louder** |
|  | **Influence of alcohol** | **PR** | **Categorical** | **No effect / Tinnitus less loud / Tinnitus louder** |
|  | **Influence of stress** | **PR** | **Categorical** | **No effect / Tinnitus less loud / Tinnitus louder** |
|  | **Influence of sleep deprivation** | **PR** | **Categorical** | **No effect / Tinnitus less loud / Tinnitus louder** |
|  | **Influence of smoking** | **PR** | **Categorical** | **No effect / Tinnitus less loud / Tinnitus louder** |
|  | **Influence of medication** | **PR** | **Categorical** | **No effect / Tinnitus less loud / Tinnitus louder** |
|  | **Influence of coffee** | **PR** | **Categorical** | **No effect / Tinnitus less loud / Tinnitus louder** |

PR: patient reported, CR: clinician reported; DD: derived data (calculated)
